# Zinc finger BED-type containing 6 (ZBED6) ameliorates cardiac fibrosis by inhibiting Piezo1 transcription and YAP nuclear translocation

**DOI:** 10.1038/s41401-025-01717-1

**Published:** 2026-01-19

**Authors:** Han Wu, Wei-tao Jiang, Qiao-yue Zhao, Xin-yue Zhang, Ping Pang, Chun-lei Wang, Zhuo Wang, Ke-ying Lin, Fang-ting Yao, Kun-kun Zou, Yu-ning Zhang, Tian-qi Duo, Feng Zhang, Ling-hua Zeng, Wei Si, Xue Kong, Jing-lun Song, Teng-fei Pan, Hong-tao Diao, Bao-feng Yang, Yu Bian

**Affiliations:** 1 Department of Pharmacology (National Key Laboratory of Frigid Zone Cardiovascular Diseases, the State-Province Key Laboratories of Biomedicine-Pharmaceutics of China, Key Laboratory of Cardiovascular Research, Ministry of Education), College of Pharmacy, Harbin Medical University, Harbin, 150081 China; 2 Affiliated Dongguan Songshan Lake Center Hospital, Guangdong Medical University, Dongguan, 523808 China; 3 The Academician Cooperative Laboratory of Basic and Translational Research on Chronic Diseases, the First Affiliated Hospital, Jinan University, Guangzhou, 510632 China; 4 Department of Pharmacology, College of Basic Medical Sciences, Jilin University, Changchun, 130021 China

**Keywords:** myocardial infarction, cardiac fibrosis, ZBED6, Piezo1, YAP

## Abstract

Cardiac fibroblasts progressively replace deceased cardiomyocytes during the development of myocardial fibrosis, an irreversible pathological repair process that ultimately leads to cardiac dysfunction and heart failure. Cardiac injury was evaluated by echocardiography and Masson staining in myocardial  infarction (MI) mice with zinc finger BED-type containing 6 (ZBED6) knockdown or overexpression. Furthermore, chromatin immunoprecipitation (ChIP) assays, electrophoretic mobility shift assays (EMSAs), and luciferase reporter assays were used to explore the target of ZBED6. ZBED6 expression was notably decreased in vivo in MI hearts and in vitro in TGF-β-induced primary mouse cardiac fibroblasts (PMCFs). Transgenic overexpression of ZBED6 specifically in cardiac fibroblasts improved cardiac dysfunction, reduced the infarct area, and decreased the expression levels of fibrotic genes after MI injury. Conversely, physiological knockdown of ZBED6 induced cardiac dysfunction and remodeling, which is consistent with the phenomena observed in vitro. Mechanistically, ZBED6, which functions as a transcriptional inhibitor of Piezo1, failed to prevent its transcription owing to mutations in the promoter binding sites. Stimulation of Piezo1 in PMCFs facilitates YAP translocation into the nucleus, whereas knockdown of Piezo1 or the use of a Piezo1 inhibitor suppresses this translocation. Moreover, the activation of Piezo1 reversed the cardioprotective effects of ZBED6 overexpression. In summary, the protective effect of ZBED6 against myocardial fibrosis injury is achieved through the inhibition of Piezo1 transcription, leading to reduced YAP nuclear translocation. These findings suggest that ZBED6 may become a potential therapeutic target for the clinical treatment of myocardial fibrosis.

## Introduction

Heart failure (HF) results in an imbalance between cardiac output and tissue metabolism and is characterized by a syndrome featuring diastolic or systolic dysfunction of the myocardium and circulatory impairment [1]. The emergence of myocardial fibrosis frequently results in the onset of HF, which is the foremost contributor to elevated global mortality rates, elevating it to the status of a paramount public health quandary on a global scale [2, 3]. This intricate and diverse cellular network comprises the cardiac tissue of mammals, in which the regenerative capacity of adult cardiomyocytes is notably limited [4, 5]. Following myocardial infarction (MI), the deficit in myocardial tissue typically yields to fibrotic substitution, engendering heightened muscular rigidity and diminished contractile efficacy within the cardiac tissue, ultimately culminating in the occurrence of cardiac dysfunction and HF [2, 6, 7]. During the progression of myocardial fibrotic diseases, cardiac tissue undergoes extensive activation of cardiac fibroblasts (CFs), followed by heightened synthesis of fibrous proteins and excessive deposition of the extracellular matrix, thereby leading to the formation of scar tissue [8, 9]. Presently, the therapeutic methods used to treat myocardial fibrosis are limited to surgical interventions, highlighting the imperative for contemporary medicine to address effective strategies targeting CFs.

Zinc finger BED-type containing 6 (ZBED6) is a highly conserved transcriptional regulator unique to placental mammals, and originates from the evolutionary adaptation of a domesticated DNA transposon that played a pivotal role in placental mammals [9, 10]. ZBED6 consists of two DNA-binding BED domains and one hobo-Ac-Tam3 dimerization domain and lacks introns, making it a member of the CCCH zinc-finger BEAT and DREF domain family [10, 11]. ZBED6 is widely distributed in mammals and is frequently reported to bind to the 5’-GCTCG-3’ motif located within the intron of insulin-like growth factor 2 (IGF2), thereby negatively regulating IGF2 transcription [10, 12]. The DNA-binding domains of ZBED6 demonstrate 100% sequence homogeneity across 26 placental mammals, suggesting its profound significance in animal growth and development [10, 13]. Pigs with mutated ZBED6 binding sites exhibit increased heart volume, reduced fat deposition, and increased muscle mass [14]. These findings highlight the pivotal role of ZBED6 within the heart, offering preliminary insights into its critical functions in cardiac physiology. However, the function of ZBED6 in heart disease remains unexplored. Similarly, ZBED6 is expressed in insulin-secreting β-cells, where it has been reported to regulate cell proliferation, apoptosis, insulin production, and reactive oxygen species generation [11]. However, whether ZBED6 participates in the process of myocardial fibrosis induced by CFs activation has not been elucidated.

The ion channel protein Piezo1 serves as a pivotal cellular mechanosensor that can perceive and respond to mechanical stimuli in the cellular environment, thereby regulating diverse cellular processes [15, 16]. A wealth of evidence indicates that Piezo1 is extensively expressed in various tissues and different cell types, including fibroblasts, cardiomyocytes, immune cells, endothelial cells, and osteoblasts [17–20]. Moreover, Piezo1 is involved in the regulation of various cellular functions, including tumor cell invasion and metastasis [21], skeletal muscle development and repair [22], and cell proliferation and differentiation [23]. Notably, Piezo1 and actomyosin activity synergistically drive the activation of urinary system fibroblasts [24], revealing the significant role of Piezo1 in regulating fibroblast activation. Previous research has confirmed that Piezo1 is upregulated in cultured cardiomyocytes and fibroblasts, suggesting that Piezo1 in the heart serves as a danger signal for ventricular remodeling, even triggering the development of HF [25–29]. Given the significance of Piezo1 in cardiac diseases and its unclear role, the mechanisms of Piezo1 in myocardial fibrosis deserve further investigation. The Hippo signaling pathway can mediate the process of cardiac injury, which is fundamental to maintaining homeostasis in the adult heart [30]. Accumulating evidence suggests that the Hippo/Yes-associated protein (YAP) pathway plays a crucial role in cardiovascular diseases, particularly in cardiovascular remodeling [31, 32]. When the Hippo pathway is inhibited, the YAP protein is translocated into the cell nucleus, where it induces gene expression and further stimulates cell proliferation and migration activity [33]. However, whether Piezo1-mediated mechanotransduction regulates YAP nuclear transport and mediates the process of myocardial fibrosis remains unclear.

In this study, we utilized MI surgery and TGF-β-activated PMCFs to provide evidence for the involvement of ZBED6 in the process of myocardial fibrosis in vivo and in vitro. We found that the transcriptional repressor ZBED6 identifies transcriptional binding sites on the Piezo1 promoter and suppresses its transcription. Piezo1 subsequently binds to YAP and promotes its nuclear translocation. Silencing Piezo1 reverses the increase in nuclear YAP protein expression induced by MI injury and TGF-β-activated PMCFs. In conclusion, our experimental findings substantiate the potential of ZBED6 as a novel therapeutic target for addressing cardiac fibrosis.

## Materials and methods

### Animals

The experiments in this study were approved by the Ethics Committee for Animal Experiments of Harbin Medical University School of Pharmacy and were conducted following the guidelines for the care and use of laboratory animals at Harbin Medical University (Approval number: IRB3026722). All the experiments were performed in accordance with the NIH Guide for the Care and Use of Laboratory Animals. Eight-week-old male C57BL/6 mice were selected as experimental subjects and housed in an environment with a humidity ranging from 30% to 70% and a temperature of 23 ± 3 °C under a 12 h light/dark cycle.

### Generation of ZBED6 transgenic mice

Transgenic mice overexpressing ZBED6 specifically in fibroblasts were generated by Cyagen Biotechnology Co., Ltd. (China). The “CAG promoter-loxP-PGK-Neo-6*SV40 pA-loxP-Kozak-Mouse Zbed6 CDS-P2A-EGFP-rBG pA” cassette was cloned and inserted into intron 1 of ROSA26, after which the DNA fragment containing ZBED6 was microinjected into fertilized eggs to generate the ZBED6^*FKI*^ mouse model. F0 founder animals, which were bred to wild-type mice to test germline transmission and F1 animal generation, were identified by qRT–PCR followed by sequence analysis. F1-targeted mice with a tissue-specific Col1a2-CreER deletion were bred to generate mice that were heterozygous for a targeted allele and hemizygous/heterozygous for the Cre transgene. ZBED6 knock-in mice were matched to Col1a2 CreER mice to generate cardiac fibroblast-specific ZBED6 knock-in mice (ZBED6^*FKI*^). Genomic DNA was isolated from tail tissue samples for qRT–PCR amplification to identify the ZBED6 genotype of offspring. All the mice were compared exclusively with sex-matched 8-week-old nontransgenic littermates.

### Mouse model of MI

The mice were anesthetized with avertin at a dosage of 0.1 mL/10 g body weight. A 7–0 silk suture was used to ligate the left anterior descending coronary artery of the left coronary artery 1–2 millimeters below the left atrium. With respect to the sham-operated mice, the suture needle only passed through, did not create a ligation; subsequently, mouse echocardiography was used to assess mouse cardiac function.

### Construction of an adeno-associated virus carrying the ZBED6 gene

The construction of periostin promoter-driven adeno-associated virus serotype 9 vectors for specific targeting of fibroblasts, both carrying the ZBED6 gene (shZBED6-V) and empty vectors (shNC-V), was commissioned from HANbio Biotechnology (Shanghai, China). AAV9 viruses were intravenously injected into 8-week-old mice via the tail vein with 1.5 × 10^11^ AAV9 ZBED6 vector genomes for 8 weeks; subsequently, mouse echocardiography was used to assess mouse cardiac function.

### Echocardiography

To evaluate the cardiac function of the experimental mice, M-mode echocardiography was performed on the mice using a Vevo2100 ultrasound system equipped with a 10 MHz phased-array transducer (VisualSonics, Toronto, Canada). M-mode traces of the left ventricle were obtained from short-axis views to record the left ventricular internal dimension at end diastole (LVID; d), left ventricular internal dimension at systole (LVID; s), ejection fraction (EF) and fractional shortening (FS). At least three consecutive cardiac cycles were recorded, and the averages were analyzed.

### Masson trichrome staining

Heart tissues were fixed in 4% paraformaldehyde, dehydrated, and subsequently embedded in paraffin. In accordance with the manufacturer’s instructions, the heart tissues were cut into 4–6 μm sections and stained with a Masson’s trichrome staining kit (Solarbio, China). The infarct area and the proportion relative to the total area were analyzed using ImageJ software.

### Western blotting

The proteins extracted from peri-infarct tissue or myofibroblasts were cleaved in RIPA cleavage buffer supplemented with 1% protease inhibitors (Roche, Switzerland). The protein concentration was determined using a bicinchoninic acid protein detection kit (Beyotime Institute of Biotechnology, Shanghai, China). The proteins were separated by sodium dodecyl sulfate–polyacrylamide gel electrophoresis and transferred to nitrocellulose membranes. The membranes were incubated with primary antibodies overnight at 4 °C. The following primary antibodies were used: anti-GAPDH (#TA-08, ZsBio, Beijing, China, 1:1000), anti-ZBED6 (#HPA068807, Atlas Antibodies, Sweden, 1:1000), and anti-Piezo1 (#15939-1-AP, Proteintech, Wuhan, China, 1:1000). The blotted membranes were scanned using an Odyssey Infrared Imaging System (LI-COR, Lincoln, NE, USA). Quantification was performed using Image Studio software, and the data were normalized to that of GAPDH as an internal control.

### QRT–PCR

RNA was extracted with TRIzol (Invitrogen, Carlsbad, CA, USA) according to the instructions provided by the manufacturer. The concentrations of RNA samples were determined using a NanoDrop ND-8000 (Thermo Fisher Scientific, Waltham, MA, USA). cDNA was subsequently synthesized using a reverse transcription kit (Toyobo, Japan) with a total RNA mass of 500 ng. The cDNA transcript products were quantified using SYBR Green (Toyobo, Japan). The mRNA expression levels were analyzed in an ABI 7900HT fast real-time PCR System (Applied Biosystems, Foster City, CA, USA) and normalized to the levels of GAPDH or 18S, after which the 2^–ΔΔCt^ method was used to calculate the amount of gene expression. The primer sequences are shown in Table S1.

### Isolation and culture of PMCFs

After anesthesia, the hearts of adult mice were removed from a superclean table, placed in PBS, cut into small pieces and treated with 50% (w/v) type II collagen and 100% (w/v) trypsin (Solarbio, Beijing, China). Digestion was then stopped, and the undigested tissue was removed by filtration through a 100 μm cell screen, followed by centrifugation to remove excess pancreatic enzymes, after which the cells were inoculated into a Petri dish.

PMCFs were extracted from neonatal mouse hearts aged 1–3 days. After being rinsed in PBS, the isolated hearts of neonatal mice were dissected into fragments and then subjected to digestion in a trypsin-EDTA solution (Solarbio, Beijing, China). The obtained cells were cultured in Dulbecco’s modified Eagle’s medium (DMEM) with 5% fetal bovine serum (Biological Industries, Haemek, Israel) and 0.8% penicillin and streptomycin (Beyotime, Shanghai, China). After culture of the cell mixture in a 37 °C incubator containing 5% CO_2_ for 2 h, the culture medium was changed, and the cells were transferred to a culture plate at the appropriate density. The cells were attached to the walls of the plate for the subsequent experiments.

### CCK8 assay

Fibroblasts were seeded in 96-well plates at 3 × 10^4^ cells per well and subjected to the required experimental treatment the next day, after which a 110 µL mixture consisting of 100 µL of DMEM (Biological Industries, Haemek, Israel) and 10 µL of CCK8 solution (Beijing Labgic Technology Co., Ltd., Beijing, China) was added to each well, followed by incubation at 37 °C for 1–2 h and selection at 450 nm in a microplate reader and measurement.

### Wound healing assay

PMCFs were seeded in 6-well plates at a density of 6 × 10^5^ cells per well. When the cell confluence reached 70%, three parallel lines were drawn vertically in each well using a sterilized 10 µL gun tip. After processing according to the experimental demands, photographs were taken at 0 and 48 h using a digital single-lens reflex camera.

### 5-Ethynyl-2′-deoxyuridine (EdU) staining

A Cell-Light™ EdU Apollo 567 In Vitro kit (RiboBio, Guangzhou, China) was used. In accordance with the manufacturer’s instructions, 3 × 10^4^ PMCFs were seeded in 24 wells. PMCFs were incubated with 50 µmol/L EdU reagent for 2 h and then were washed with PBS. After being fixed and decolored, the cells were permeabilized with 300 µL of 0.5% Triton X-100. Next, Apollo and Hoechst 33342 stains were used, and the images were observed and recorded by a fluorescence microscope.

### Immunofluorescence

After heart paraffin sections were dewaxed or treated with PMCFs inoculated in a 24-well glass substrate, the sections were washed with PBS, treated with 0.1% Triton X-100, and penetrated for 10 min. Afterward, 5% goat serum was used to block the sections for 30 min. The primary antibody (dilution ratio 1:200) was incubated at 4 °C overnight. After being washed with PBS, the sections were treated again with 0.1% Triton X-100 and then incubated at room temperature for 30 min with a secondary antibody (dilution ratio 1:500). The nuclei were stained with DAPI, and the images were observed and recorded using a laser scanning confocal microscope.

### Chromatin immunoprecipitation (ChIP) assay

After transfection with ZBED6 overexpression plasmids, ChIP detection was performed according to the standard protocol provided for the Pierce™ Agarose ChIP Kit (Thermo Scientific, Carlsbad, CA, USA). Briefly, the cells were crosslinked with formaldehyde and then lysed with lysis buffer. Afterward, fragments of 150–900 bp obtained by ultrasonicating chromatin were incubated with ZBED6 antibody or isotype-matched control IgG at 4 °C overnight. The immune complexes were subsequently eluted and purified. DNA was subjected to further analysis by qRT–PCR.

### EMSA (electrophoretic mobility shift assay)

A LightShift™ chemiluminescent EMSA kit (Thermo Fisher Scientific, Waltham, USA) was used for RNA electrophoretic mobility change analysis. In accordance with the manufacturer’s instructions, nuclear extract was extracted with binding buffer and a labeled probe, poly (dI. Dc) for 30 min at 20 °C. The complexes were decomposed on a polyacrylamide gel by incubation with mutant probes or excessive amounts of unlabeled cold competitive probes and then cross-linked and detected by chemiluminescence. The biotin-labeled probe sequence was 5’-GTAGACCAGGCTGGCCTCGAACTC-biotin 3’; the sequence of the cold competitive probes was 5’- GTAGACCAGGCTGGCCTCGAACTC-3’; and the sequence of the mutant probe was 5′-GTAGACACTTAGTTAAGAGAACTC-3’.

### Luciferase reporter assays

Luciferase activity was analyzed using a dual-luciferase reporter assay system (Promega, Wisconsin, USA). In accordance with the manufacturer’s instructions, the designed Piezo1 promoter plasmids were transfected into cells with transfection reagent. Luciferase activity was determined by measuring the ratio of firefly to Renilla luciferase activity.

### Statistical analysis

The data were statistically analyzed using GraphPad Prism 7.0. The results are expressed as the mean ± SEM. Comparisons between two groups were made using Student’s *t* test. Multiple groups were compared using one-way ANOVA with Dunnett’s correction. A *P* value < 0.05 was considered to indicate statistical significance.

## Results

### The expression of ZBED6 is downregulated in the injured myocardium induced by MI and in TGF-β-activated PMCFs

**Fig. 1:**
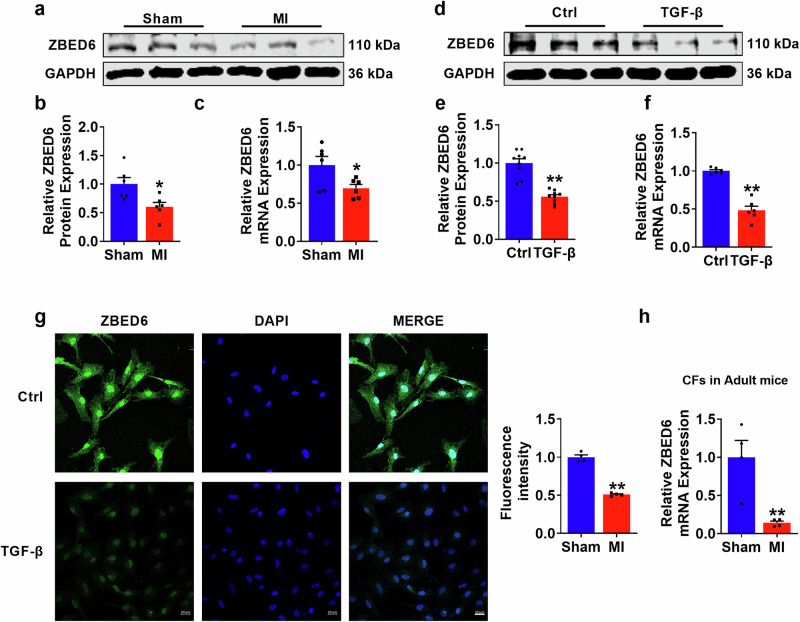
ZBED6 expression levels in MI-4 week-mice and TGF-β activated PMCFs. **a**–**c** ZBED6 mRNA and protein levels in MI mice. *n* = 6. ^*^P < 0.05 vs. Sham. **d**–**f** The mRNA and protein levels of ZBED6 in TGF-β treated PMCFs. *n* = 6–9. ^**^P < 0.01 vs. Ctrl. **g** Representative fluorescent diagram of ZBED6 expression in PMCFs. Scale bar = 20 μm. *n* = 4. ^**^P < 0.01 vs. Sham. **h** The expression level of ZBED6 in CFs obtained from male adult C57BL/6 mice. *n* = 4. ^**^P < 0.01 vs. Sham.

To elucidate the regulation of ZBED6 in MI-induced cardiac fibrosis, we systematically examined its expression in fibrotic models. ZBED6 mRNA and protein expression levels were significantly decreased in mice following MI (Fig. 1a–c). To confirm the cellular localization of ZBED6, we used the Human Protein ATLAS database, which revealed that the increased expression of ZBED6 in cardiac myofibroblasts in 4 human heart samples was close to half of the expression in the entire cardiac tissue sample (Supplementary Fig. S1a). Similarly, the in vitro results demonstrated significant decreases in the mRNA and protein expression levels of ZBED6 in TGF-β-induced PMCFs isolated from neonatal mice (Fig. 1d–f). The immunofluorescence data revealed the same results, which were consistent with the observation that ZBED6, a transcription factor, is predominantly localized in the nuclei of PMCFs (Fig. 1g). Moreover, we isolated CFs from Sham and MI-4 week-mice and found that ZBED6 expression was significantly decreased in myofibroblasts extracted from MI mice (Fig. 1h).

### Transgenic overexpression of ZBED6 alleviates cardiac fibrosis injury in mice

**Fig. 2:**
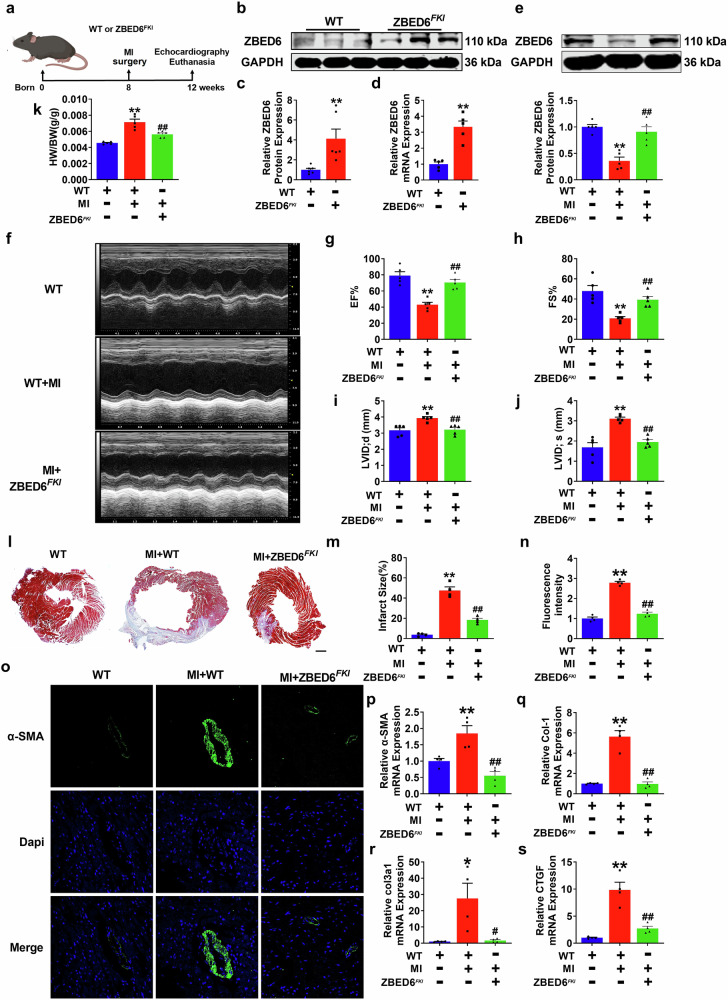
ZBED6 transgenic overexpression reverses cardiac fibrosis induced by MI in mice. **a** A schematic diagram of experimental design. **b**–**d** ZBED6 protein and mRNA levels in ZBED6 transgenic mice hearts. *n* = 5–6. ^**^P < 0.01 vs. WT. **e** The representative Western blot diagram of ZBED6 was shown. *n* = 5. ^**^P < 0.01 vs. WT; ^##^*P* < 0.01 vs. MI + WT. **f** Echocardiography images in each group. **g** EF (%), (**h**) FS (%), (**i**) LVID; d (mm) and **j** LVID; s (mm). *n* = 5. ^**^P < 0.01 vs. WT; ^##^*P* < 0.01 vs^.^ MI + WT. **k** The ratio of heart weight to body weight. *n* = 5. ^**^P < 0.01 vs. WT; ^##^*P* < 0.01 vs. MI + WT. **l** Infarcted area of mice hearts by Masson staining. Scale bar = 1 mm. *n* = 4. **m** The area statistics of the Masson staining. *n* = 4. ^**^P < 0.01 vs. WT; ^##^*P* < 0.01 vs. MI + WT. **n**, **o** Immunofluorescence expression of α-SMA in mouse hearts. Scale bar = 20 μm. *n* = 4. ^**^P < 0.01 vs. WT; ^##^*P* < 0.01 vs. MI + WT. **p**–**s** Fibrotic gene markers in each group. *n* = 4. ^*^P < 0.05, ^**^P < 0.01 vs. WT; ^#^*P* < 0.05, ^##^*P* < 0.01 vs. MI + WT.

To elucidate the functional role of ZBED6 in cardiac fibrosis injury, we generated transgenic mice (Supplementary Fig. S2a) with fibroblast-specific overexpression of ZBED6 (ZBED6^*FKI*^), which is driven by the periostin promoter, and established an MI injury model (Fig. 2a). ZBED6 protein and mRNA expression levels were significantly increased by nearly four-fold in the heart tissues of ZBED6^*FKI*^ mice compared with those of wild-type controls (Fig. 2b–d). In addition, ZBED6^*FKI*^ markedly reversed the decrease in ZBED6 expression caused by MI injury in mice (Fig. 2e). The echocardiographic test results revealed that overexpression of ZBED6 effectively improved cardiac dysfunction, which was reflected by the increased EF and FS and decreased LVID; d and LVID; s (Fig. 2f–j). The heart function of wild-type mice was not affected (Supplementary Fig. S3a–e). Moreover, the heart weight-to-body weight ratio and fibrotic area were reduced in ZBED6 transgenic mice after MI surgery (Fig. 2k–m), but these parameters were not affected at the physiological level (Supplementary Fig. S3f–h). Immunofluorescence staining revealed that ZBED6 overexpression decreased α-SMA fluorescence intensity (Fig. 2n, o). Consistent with these findings, the abnormal increase in the expression of fibrotic genes was restrained by ZBED6 overexpression (Fig. 2p–s, Supplementary Fig. S4a, b). These data demonstrate that ZBED6 protects against cardiac fibrosis induced by MI injury.

### Deletion of ZBED6 in myofibroblasts facilitates cardiac dysfunction and adverse ventricular remodeling

**Fig. 3:**
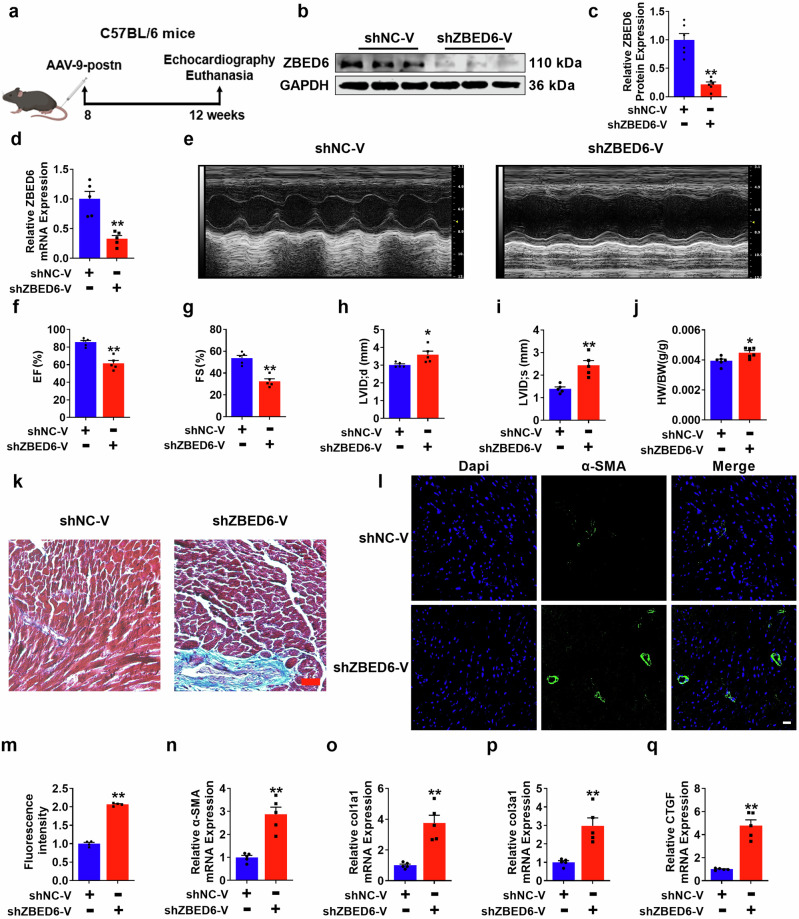
ZBED6 silencing exacerbates cardiac fibrosis. **a** A schematic diagram of experimental design. **b**–**d** The expression of ZBED6 in ZBED6 silencing mice (shZBED6-V) and control mice (shNC-V). *n* = 5–6. ^**^P < 0.01 vs. shNC-V. **e** Echocardiographs and statistics. **f** EF (%), **g** FS (%), **h** LVID;d (mm) and **i** LVID;s (mm). *n* = 5. ^*^P < 0.05, ^**^P < 0.01 vs. shNC-V; **j** The heart weight to body weight ratio in the two groups. *n* = 6. ^*^P < 0.05 vs. shNC-V. **k** Representative images of Masson staining in ZBED6 knockdown mice. Scale bar = 50 μm. *n* = 4. **l, m** Immunofluorescence diagram of α-SMA in mouse hearts. Scale bar = 20 μm. *n* = 4. ^**^P < 0.01 vs. shNC-V. **n**–**q** The mRNA expression levels of fibrosis-related genes in mice. *n* = 5. ^**^P < 0.01 vs. shNC-V.

Following prolonged cardiac injury, myofibroblasts transformation plays a crucial role during the healing phase. Nevertheless, excessive activation of myofibroblasts can also lead to impaired cardiac function and pathological ventricular remodeling [34]. To thoroughly investigate the role of ZBED6 in the functionality of myofibroblasts, we used periostin promoter-driven AAV9 carrying shZBED6-V to knock down endogenous ZBED6 in the myofibroblasts of mouse hearts (Fig. 3a). First, we used qRT–PCR and Western blotting to confirm the successful knockdown of ZBED6 in heart tissue (Fig. 3b–d). Deletion of ZBED6 significantly impaired cardiac function, as manifested by decreased EF and FS and elevated LVID; d and LVID; s compared with those of the Sham mice (Fig. 3e–i). Following intravenous tail injection of the virus for 8 weeks, ZBED6 knockdown increased the heart-to-body weight ratio in mice (Fig. 3j). As depicted in the data in Fig. 3k, compared with the control, ZBED6 silencing resulted in increased collagen deposition. Consistent with previous results, the expression levels of fibrotic markers, including α-SMA, Col1a1, Col3a1, and CTGF, significantly increased following ZBED6 silencing (Fig. 3l–q, Supplementary Fig. S5a, b). Taken together, the above findings suggest that the absence of ZBED6 in mice results in adverse effects, including impaired cardiac function and myocardial fibrosis.

### Regulatory role of ZBED6 in mediating TGF-β-induced myofibroblast activation

**Fig. 4:**
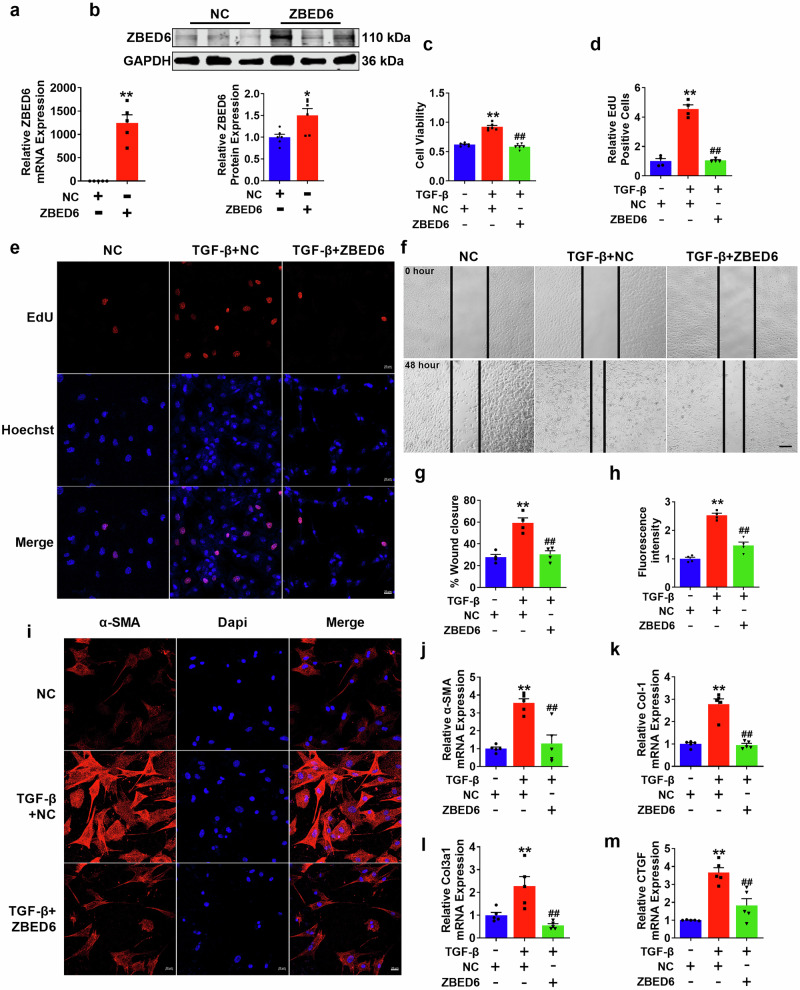
ZBED6 overexpression prevents PMCFs activation induced by TGF-β in vitro. **a**, **b** ZBED6 overexpression plasmid transfection efficiency was verified by qRT-PCR and Western blot. *n* = 5–6. ^*^P < 0.05, ^**^P < 0.01 vs. NC. **c** Cell viability measured by CCK8 in each group. *n* = 6. ^**^P < 0.01 vs. NC; ^*##*^*P *< 0.01 vs. TGF-β + NC. **d**, **e** The proliferation of PMCFs was detected by EdU staining. *n* = 4. ^**^P < 0.01 vs. NC; ^*##*^*P* < 0.01 vs. TGF-β + NC **f**, **g** The migration ratio in each group. Scale bar = 100 μm. *n* = 4. ^**^P < 0.01 vs. NC; ^*##*^*P* < 0.01 vs. TGF-β + NC. **h**, **i** Fluorescence intensity of α-SMA in PMCFs. Scale bar = 20 μm. *n* = 4. ^**^P < 0.01 vs. NC; ^*##*^*P* < 0.01 vs. TGF-β + NC. **j**–**m** The fibrotic genes mRNA expression levels. *n* = 5. ^**^P < 0.01 vs. NC; ^*##*^*P* < 0.01 vs. TGF-β + NC.

To further investigate the role of ZBED6 in vitro, we investigated the impact of ZBED6 overexpression on the TGF-β-induced activation of PMCFs. Following transfection with ZBED6, both the mRNA and protein expression levels of ZBED6 significantly increased (Fig. 4a, b). Additionally, our results indicated that the cell viability of myofibroblasts, proliferation count, and migration rate significantly increased after treatment with TGF-β; however, this aberrant phenomenon could be suppressed by ZBED6 (Fig. 4c–g). Immunofluorescence experiments revealed that ZBED6 reduced the pathological increase in α-SMA fluorescence intensity following TGF-β stimulation in PMCFs (Fig. 4h, i). In addition, the expression levels of fibrosis-related marker genes in activated PMCFs significantly decreased after ZBED6 was overexpressed (Fig. 4j–m, Supplementary Fig. S6a, b). These results provide compelling evidence for the protective role of ZBED6 in regulating abnormal proliferation and migration in response to TGF-β-induced activation of PMCFs in vitro.

### PMCFs activation is modulated by the absence of ZBED6 in vitro

**Fig. 5:**
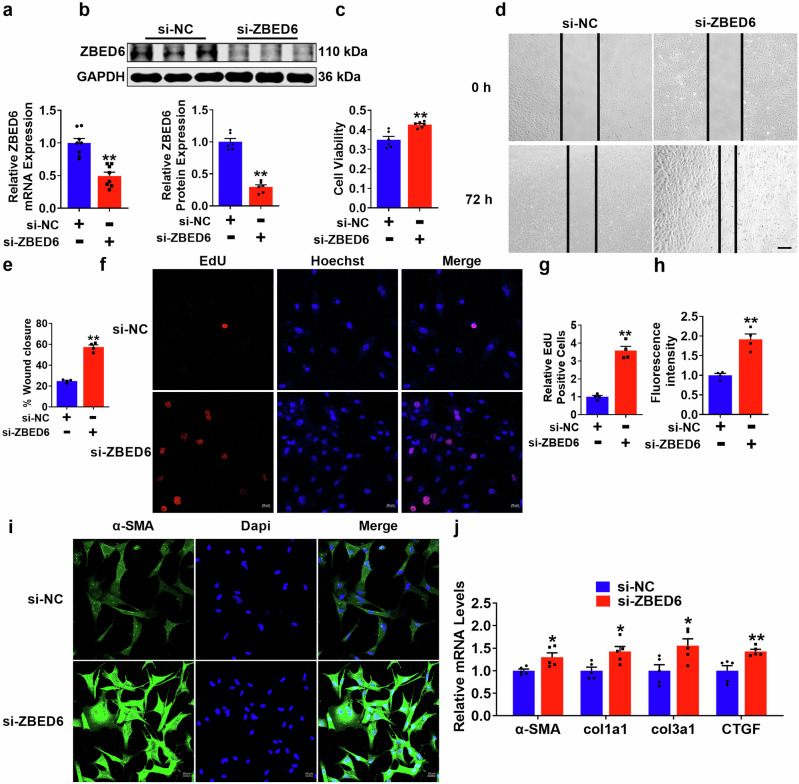
The absence of ZBED6 regulated PMCFs activation. **a**, **b** ZBED6 silencing efficiency proved by qRT-PCR and Western blot. *n* = 6–8. ^**^P < 0.01 vs. si-NC. **c** CCK-8 detected PMCFs viability. *n* = 6. ^**^P < 0.01 vs. si-NC. **d**, **e** PMCFs migration ratio after silencing ZBED6. Scale bar = 100 μm. *n* = 4. ^**^P < 0.01 vs. si-NC. **f**, **g** EdU assay of PMCFs proliferation. *n* = 4. ^**^P < 0.01 vs. si-NC. **h**, **i** Representative immunofluorescence diagram of α-SMA. Scale bar = 20 μm. *n* = 4. ^**^P < 0.01 vs. si-NC. **j** The fibrotic genes mRNA expression. *n* = 5. ^*^P < 0.05, ^**^P < 0.01 vs. si-NC.

We subsequently focused our investigation on elucidating the regulatory impact of ZBED6 deficiency on the activation of PMCFs. We constructed ZBED6 siRNA to silence endogenous ZBED6 expression in PMCFs, and the results revealed that transfection with si-ZBED6 partially reduced the expression of ZBED6 (Fig. 5a, b). The loss of ZBED6 increased the viability of the PMCFs, as confirmed by the results of the CCK8 assay (Fig. 5c), promoted the migration of the PMCFs, as demonstrated by the results of the wound healing experiments (Fig. 5d, e), and increased the proliferation of the PMCFs, as verified by EdU staining (Fig. 5f, g). Moreover, the fluorescence intensity of α-SMA also increased after ZBED6 silencing (Fig. 5h, i). In addition, qRT–PCR analysis revealed that ZBED6 knockdown increased the expression levels of fibrotic genes (Fig. 5j, Supplementary Fig. S7a, b). These findings suggest that the absence of ZBED6 affects the activity of PMCFs, thereby promoting the progression of cardiac fibrosis in vitro.

### ZBED6 inhibits the transcription of Piezo1

**Fig. 6:**
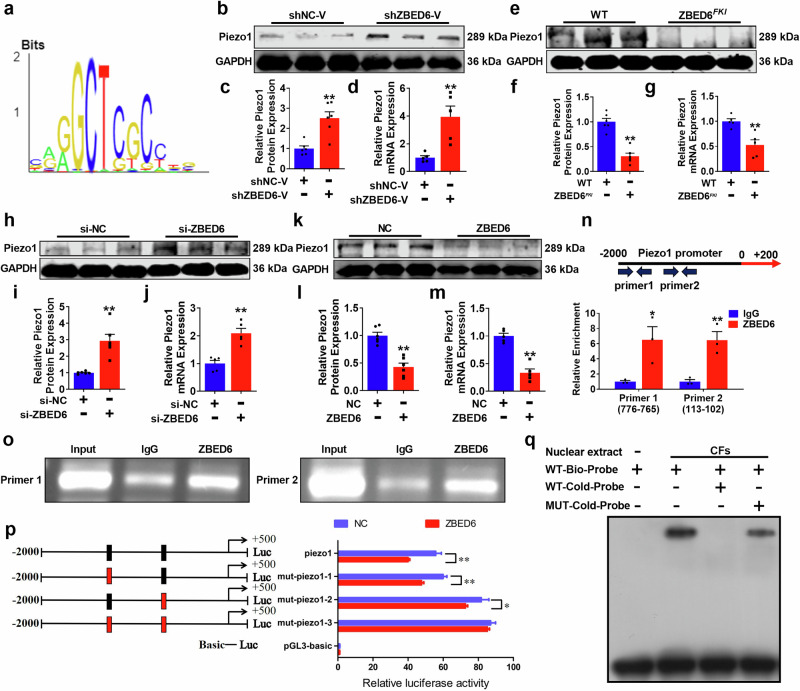
ZBED6 inhibits Piezo1 transcription. **a** The prediction of the potential binding sequence between ZBED6 and Piezo1 promoter region. **b–d** Piezo1 expression level after silencing ZBED6. *n* = 5–6. ^**^P < 0.01 vs. shNC-V. **e**–**g** The expression level of Piezo1 in ZBED6^*FKI*^ mice compared with WT mice. *n* = 5–6. ^**^P < 0.01 vs. WT. **h**–**j** Piezo1 expression level was verified by qRT-PCR and Western blot in vitro. *n* = 5–6. ^**^P < 0.01 vs. si-NC. **k**–**m** The expression level of Piezo1 in ZBED6 overexpression and NC group. *n* = 5–6. ^**^P < 0.01 vs. NC. **n** Piezo1 enrichment detected by ChIP assay. *n* = 3. ^*^P < 0.05, ^**^P < 0.01 vs. IgG. **o** Agarose gel electrophoresis representative image. **p** The relative activity of luciferase. *n* = 3. ^*^P < 0.05, ^**^P < 0.01 vs. NC. **q** The binding between ZBED6 and Piezo1 promoter detected by EMSA assay.

To further elucidate the protective mechanism of the transcription inhibitor ZBED6 in MI injury, the Transcription Factor Target Gene Database (TFBS-Home (systemsbiology.net)) was used to predict the downstream genes regulated by ZBED6 (Fig. 6a). We identified Piezo1 as a gene regulated by ZBED6 and verified this hypothesis experimentally (Supplementary Fig. S8). First, we detected Piezo1 protein expression levels in the three groups and found that Piezo1 expression was upregulated in MI mice, whereas ZBED6 overexpression inhibited its expression (Supplementary Fig. S9a, b). More convincingly, ZBED6 overexpression or silencing decreased or increased, respectively, the mRNA and protein expression levels of Piezo1 both in vivo and in vitro (Fig. 6b–m). We subsequently used the AnimalTFDB v4.0 (AnimalTFDB4 (wchscu.cn)) website to predict the potential binding sites of the transcription factor ZBED6 to initiate Piezo1 transcription, and the results revealed two putative binding sites in the Piezo1 promoter (Fig. 6n). Next, we designed two distinct pairs of different primers for the binding site to perform ChIP and ChIP–qPCR experiments, and the results confirmed the interaction between ZBED6 and the Piezo1 promoter (Fig. 6n, o). Moreover, the results of dual-luciferase reporter gene experiments and EMSA analysis further confirmed the mechanism through which ZBED6 regulates Piezo1 (Fig. 6p, q). These data indicate that ZBED6 acts as a negative transcription regulator to control the transcription of Piezo1.

### Piezo1 mediates the regulatory effect of ZBED6 on PMCFs activation

**Fig. 7:**
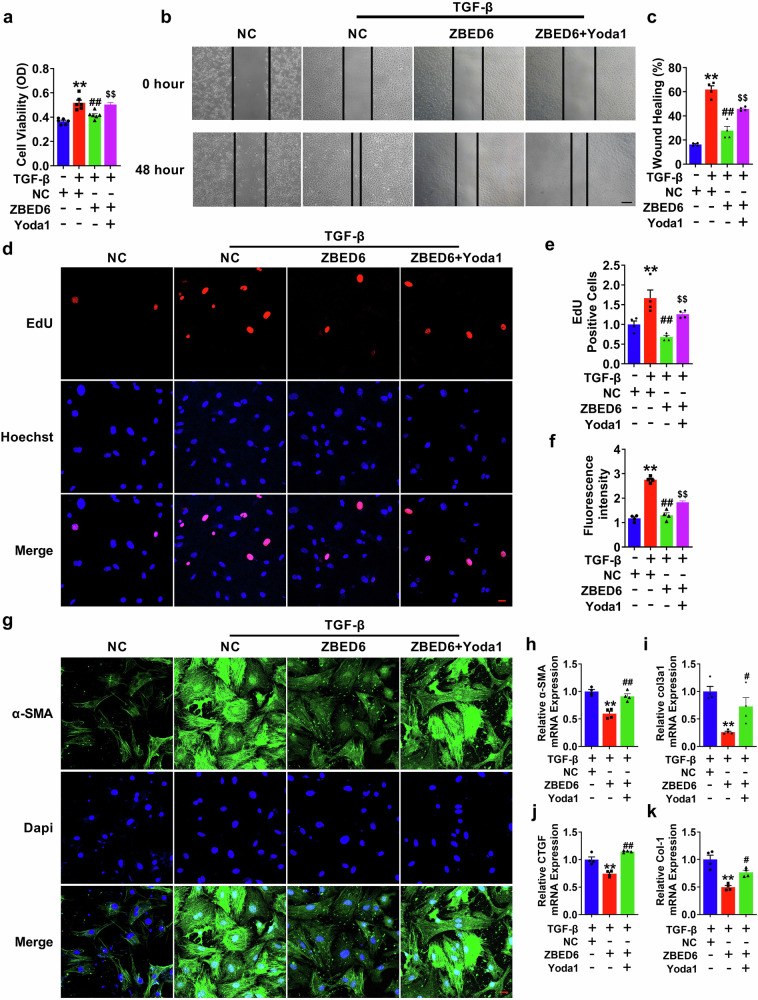
Piezo1 mediates the regulation of ZBED6 on PMCFs activation. **a** The vitality of PMCFs detected by CCK8 assay. *n* = 6. ^**^P < 0.01 vs. NC; ^##^*P* < 0.01 vs. TGF-β + NC; ^$$^*P* < 0.01 vs. TGF-β + ZBED6. **b**, **c** PMCFs migration ratio after activated by Yoda1. Scale bar = 100 μm. *n* = 4. ^**^P < 0.01 vs. NC; ^##^*P* < 0.01 vs. TGF-β + NC; ^$$^*P* < 0.01 vs. TGF-β + ZBED6. **d**, **e** The ability of PMCFs proliferation was detected by EdU. *n* = 4. ^**^P < 0.01 vs. NC; ^##^*P* < 0.01 vs. TGF-β + NC; ^$$^*P* < 0.01 vs. TGF-β + ZBED6. **f**, **g** Expression of α-SMA in PMCFs determined by immunofluorescence. Scale bar = 20 μm. *n* = 4. ^**^P < 0.01 vs. NC; ^##^*P* < 0.01 vs. TGF-β + NC; ^$$^*P* < 0.01 vs. TGF-β + ZBED6. **h–k** Fibrotic genes mRNA expression levels measured by qRT-PCR. *n* = 4. ^**^P < 0.01 vs. TGF-β + NC^; #^*P* < 0.05, ^##^*P* < 0.01 vs. TGF-β + ZBED6.

Next, we verified the functional role of ZBED6 mediated by Piezo1 in myocardial fibrosis. CCK8 and EdU assays confirmed that the Piezo1 agonist Yoda1 reversed the inhibitory effect of ZBED6 on PMCFs proliferation. In addition, Yoda1 stopped the protective effect of ZBED6 on migration induced by TGF-β (Fig. 7a–e). Furthermore, our data revealed that after treatment with TGF-β and NC, PMCFs could secrete more collagen and produce more fibrotic genes (including α-SMA, Col1a1, Col3a1, and CTGF), effects that were alleviated by ZBED6 overexpression, whereas the addition of Yoda1 abolished these protective effects (Fig. 7f–k). Taken together, these data indicate that ZBED6 inhibits cardiac fibroblast activation through the suppression of Piezo1.

### Piezo1 facilitates YAP translocation to the nucleus to participate in fibrosis

**Fig. 8:**
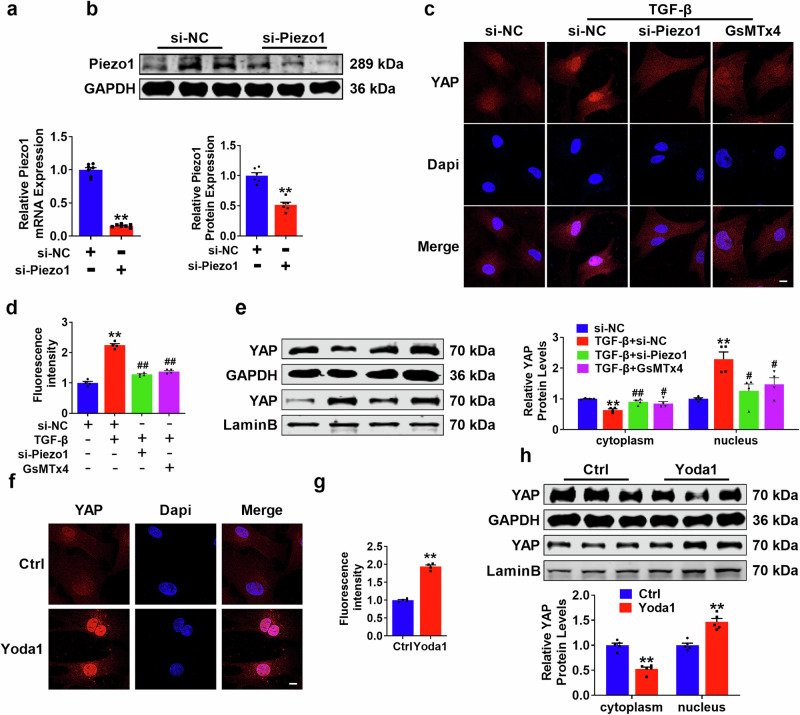
Piezo1 facilitates YAP translocation to the nucleus. **a**, **b** Piezo1 knockdown efficiency verified by qRT-PCR and Western blot. *n* = 6–7. ^**^P < 0.01 vs. si-NC. **c**, **d** Distribution of YAP displayed by fluorescent diagram. Scale bar = 20 μm. *n* = 4. ^**^P < 0.01 vs. si-NC; ^##^*P* < 0.01 vs. TGF-β+si-NC. **e** Relative expression of  YAP in nucleus and cytoplasm verified by Western blot. *n* = 4. ^**^P < 0.01 vs. si-NC; ^#^*P* < 0.05, ^##^*P* < 0.01 vs. TGF-β+si-NC. **f**, **g** YAP nucleoplasm distribution in PMCFs. Scale bar = 20 μm. *n* = 4. ^**^P < 0.01 vs. Ctrl. **h** YAP expression level in nucleus and cytoplasm. *n* = 5. ^**^P < 0.01 vs. Ctrl.

To explore the mechanism through which Piezo1 regulates cardiac fibrosis, we used Piezo1-specific knockdown siRNA to knock down Piezo1 and verified its efficiency (Fig. 8a, b). The results of immunofluorescence experiments confirmed that YAP was transferred to the nucleus in large quantities under the induction of TGF-β, and the distribution could be reversed by silencing Piezo1, whose inhibitory effect was similar to that of the Piezo1 inhibitor GsMTx4 (Fig. 8c, d). In addition, silencing Piezo1 and GsMTx4 decreased YAP protein expression in the nuclear fraction but increased it in the cytoplasmic fraction (Fig. 8e). In contrast, when Piezo1 was activated, the movement of YAP from the cytoplasm to the nucleus increased (Fig. 8f–h). These results confirm that Piezo1 targets YAP and promotes its transport to the nucleus.

## Discussion

**Fig. 9:**
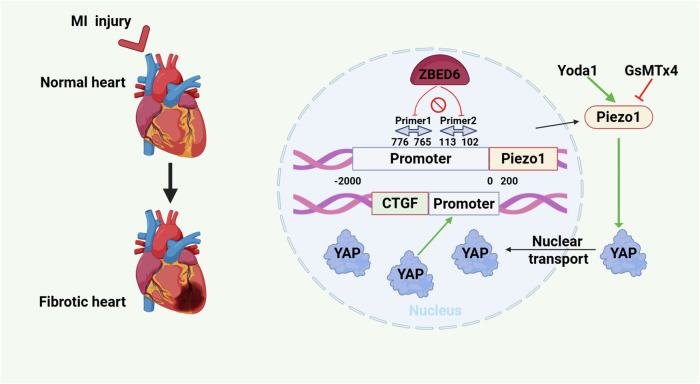
Schematic diagram illustrating the mechanism by which ZBED6 attenuates cardiac fibrosis. The transcriptional repressor ZBED6 inhibits the transcription of Piezo1, which blocks the nuclear translocation of YAP and its pro-fibrotic activity.

In this study, we report for the first time that ZBED6 expression is decreased in MI injury and activated PMCFs induced by TGF-β and that ZBED6 exerts a protective effect against myocardial fibrosis by improving cardiac function and reducing the fibrotic area to alleviate overall myocardial fibrosis. Our experimental results confirmed that ZBED6 directly binds to “CAGGCTGGCCTC” sites on the Piezo1 promoter and suppresses the transcription of Piezo1, a pernicious molecule involved in cardiac fibrosis injury, to inhibit the nuclear translocation of YAP. A lack of ZBED6 alone could lead to a fibrotic phenotype, whereas overexpression of ZBED6 could alleviate the fibrotic lesions caused by MI damage. Mechanistically, the harmful effect of YAP nuclear translocation was mediated by Piezo1 in PMCFs (Fig. 9). Moreover, for the first time, we provide strong evidence that Piezo1-specific agonists and inhibitors regulate YAP nuclear translocation (Fig. 8). This discovery provided us with an innovative understanding of the function of ZBED6 in the heart and suggested that ZBED6 could be a potential therapeutic target for treating myocardial fibrosis.

Cardiac fibrosis is a hallmark pathological response in diseases related to cardiac remodeling [35, 36]. Conditions such as MI, diabetes, and cardiomyopathy can induce cardiac fibrosis, and prolonged cardiac pressure can eventually lead to HF [37]. ZBED6 has a broad tissue distribution, influencing heart size, muscle growth, and fat deposition [10, 14]. However, the functional role of ZBED6 in the heart has not been thoroughly explored. ZBED6 has been extensively reported to be a transcriptional suppressor of IGF2, thereby regulating the growth of skeletal muscle and internal organs [38]. However, the ability of ZBED6 loss or gain to modulate the pathological process of cardiac fibrosis caused by long-term MI injury remains incompletely understood. In this study, the role of ZBED6 itself in myocardial fibrosis was further explored, and we discovered that ZBED6 mRNA and protein expression levels were downregulated in MI injury and activated PMCFs. We found that fibroblast-specific knockdown of ZBED6 alone caused cardiac function damage and increased the fibrotic area in mice, which is consistent with previous reports that ZBED6-knockout-lean Bama miniature pigs have significantly larger muscle fiber areas and heavier heart organs [39]. Conversely, ZBED6 overexpression in transgenic mice mitigated fibrotic damage and improved cardiac function resulting from MI injury. These findings indicate that ZBED6 is a key regulator of myocardial fibrosis in vivo. In vitro, excessive proliferation and activation of PMCFs can lead to scar formation, causing irreversible damage to the heart [40]. In this study, we focused on CFs and explored the function of ZBED6. Additionally, in vitro, ZBED6 silencing alone promoted the proliferation and migration of fibroblasts, and the expression levels of fibrosis-related genes also significantly increased in PMCFs. These results indicate that ZBED6 plays an important regulatory role in myocardial fibrosis.

A hypertension study revealed that GPR146 upregulates Piezo1 expression via cAMP–CREB1 signaling in vascular smooth muscle cells, driving hypertension and vascular remodeling. Piezo1 deletion prevents GPR146-induced vascular dysfunction, whereas GPR146 neutralization alleviates symptoms, highlighting the key role of Piezo1 in hypertensive pathology [41]. Another study demonstrates that Piezo1 knockout prevents hypoxic PH progression, reduces pulmonary hypertension and preserves capillaries by mitigating endothelial senescence [42]. These findings highlight the therapeutic potential of Piezo1. More importantly, the mechanosensitive ion channel Piezo1 is capable of converting various mechanical forces into intracellular signals, leading to the development of HF [43]. During HF, Piezo1 is involved in the repair processes of various cells, including endothelial cells, fibroblasts, and cardiomyocytes [27, 44]. Many studies have reported that Piezo1 expression is upregulated in cardiomyocytes and fibroblasts in the heart after they are subjected to pressure [26–29]. More importantly, thoracic dorsal root ganglion neuron-derived Piezo1 triggers neurogenic inflammation, which plays a critical role in the development of ventricular remodeling in MI mice [45]. These findings may indicate that Piezo1 plays an important role as a damage factor in cardiac ventricular remodeling. In this study, we predicted the downstream gene dataset regulated by ZBED6 using a website platform and identified upregulated Piezo1 in injury as a key regulatory factor in myocardial infarction-related lesions. The expression level of Piezo1 in the infarcted area was significantly greater in MI mice than in Sham mice. The results of ZBED6 transgenic overexpression experiments demonstrated that ZBED6 can significantly inhibit Piezo1 transcription, exerting a protective effect against cardiac fibrosis. Multiple studies have reported that YAP plays a critical role in the fibrotic progression of various fibrotic diseases, including left ventricular remodeling after MI, providing a strong theoretical basis for potential antifibrotic therapies based on fibroblasts [46–48]. The activation of Piezo1 can induce transient Ca^2+^ influx, facilitating the nuclear translocation of the transcriptional coactivator YAP and leading to the differentiation of neurons and glial cells [49].

Additionally, activation of the Piezo1-mediated mechanical pathway is involved in the development of myocardial fibrosis following MI injury. In our study, silencing Piezo1 and using a Piezo1 inhibitor (GsMTx4) mitigated the transport of YAP from the cytoplasm to the nucleus to protect against cardiac fibrotic injury, whereas the Piezo1 agonist Yoda1 promoted this adverse phenomenon. Moreover, we found that Yoda1 reversed the inhibitory effect of ZBED6 overexpression on the proliferation, migration, and expression of fibrotic genes (α-SMA, Col1a1, Col3a1, and CTGF) in activated PMCFs. These results suggest that Piezo1 can activate PMCFs via the YAP pathway, thereby influencing fibrosis.

In conclusion, we revealed for the first time that ZBED6 is a critical regulator of myocardial fibrosis injury; ZBED6 inhibits the expression of Piezo1 by inhibiting its transcription and subsequently prevents the nuclear translocation of YAP, thus promoting its function in the heart. These findings provide new insights into ZBED6 function in the heart and suggest that ZBED6 may become a potential target for the prevention and treatment of myocardial fibrosis.

## Supplementary information

Supplementary information
