# Supplementary material for: Zinc finger BED-type containing 6 (ZBED6) ameliorates cardiac fibrosis by inhibiting Piezo1 transcription and YAP nuclear translocation
Source: Acta Pharmacol Sin. 2026 Jan 19;47(5):1162–75. doi: 10.1038/s41401-025-01717-1 (PMC13109400; doi:10.1038/s41401-025-01717-1)
Supplement: Supplementary file 1 — Supplementary information [file 41401_2025_1717_MOESM1_ESM.docx]

**Supplementary materials**

**Supplementary figures and figure legends**

**Figures S1–S9**


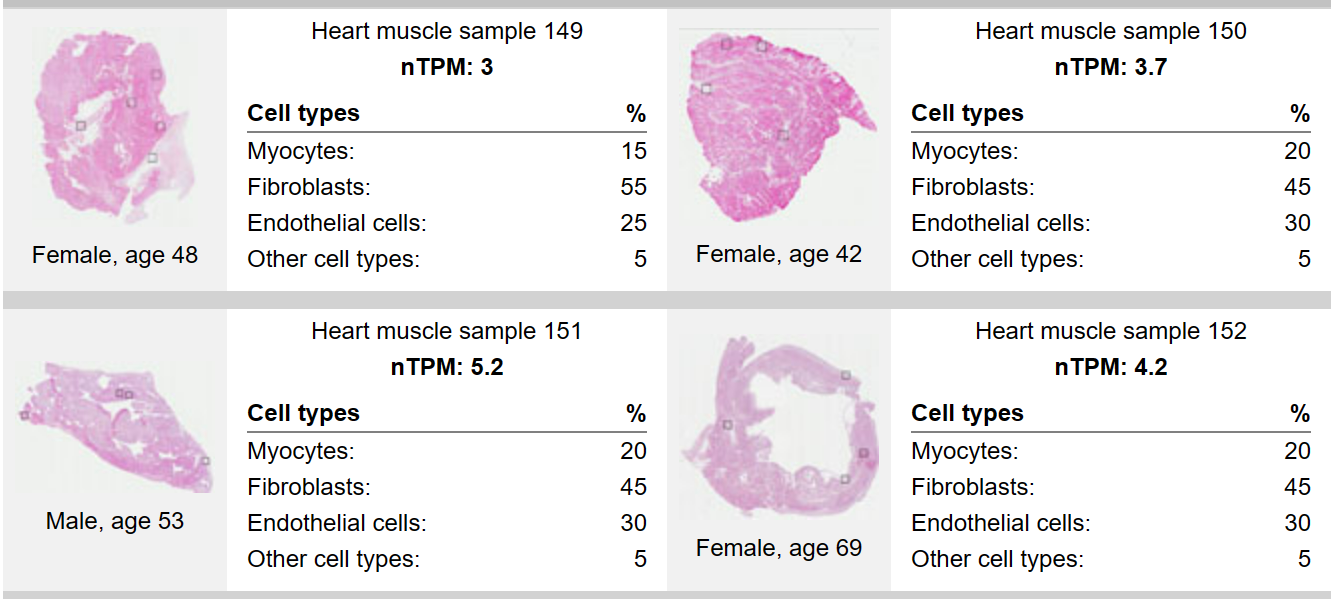


**Fig. S1.** The human protein ATLAS database analyzes the expression of ZBED6 in various cardiac cells.

**
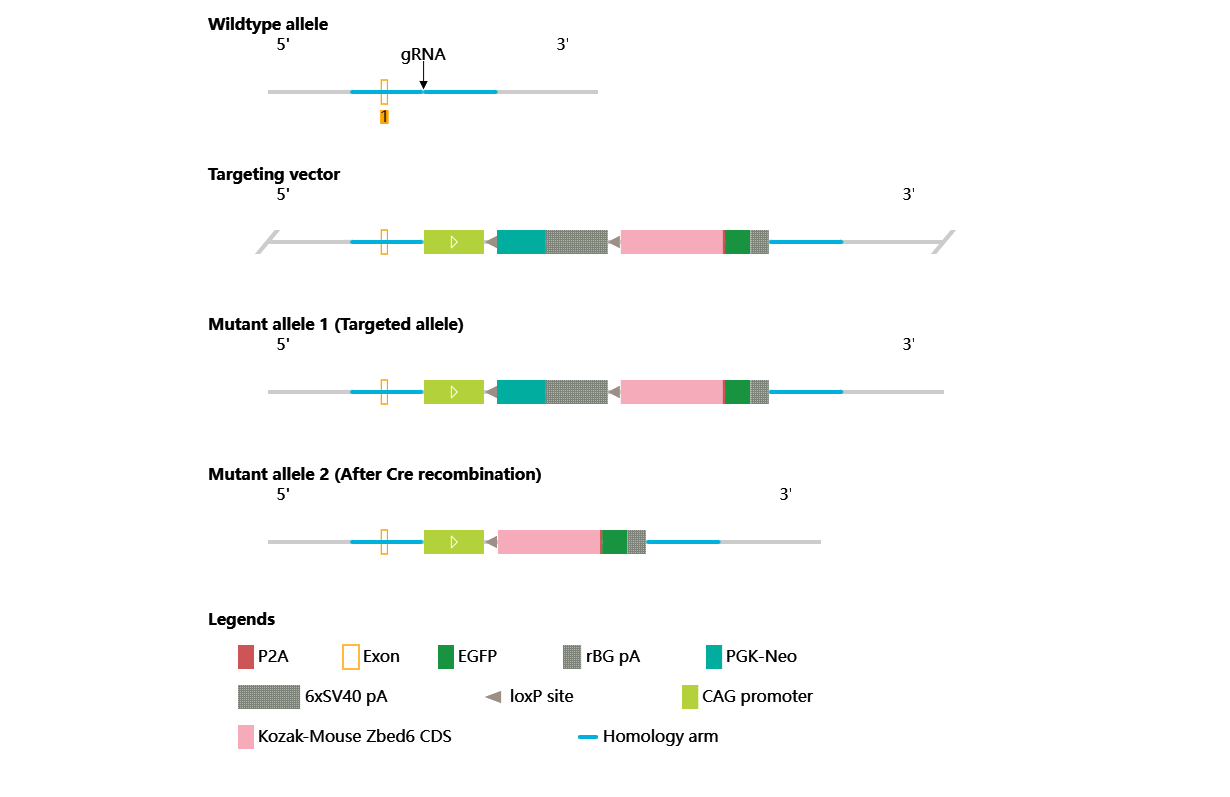
**

**Fig. S2.** The cardiac-specific ZBED6 TG mice. “CAG promoter-loxP-PGK-Neo-6*SV40 pA-loxP-Kozak-Mouse Zbed6 CDS-P2A-EGFP-rBG pA” cassette will be cloned into intron 1 of ROSA26, then microinjecting of the DNA fragment containing ZBED6 into fertilized eggs to generate the ZBED6 (TG) mouse model.

**
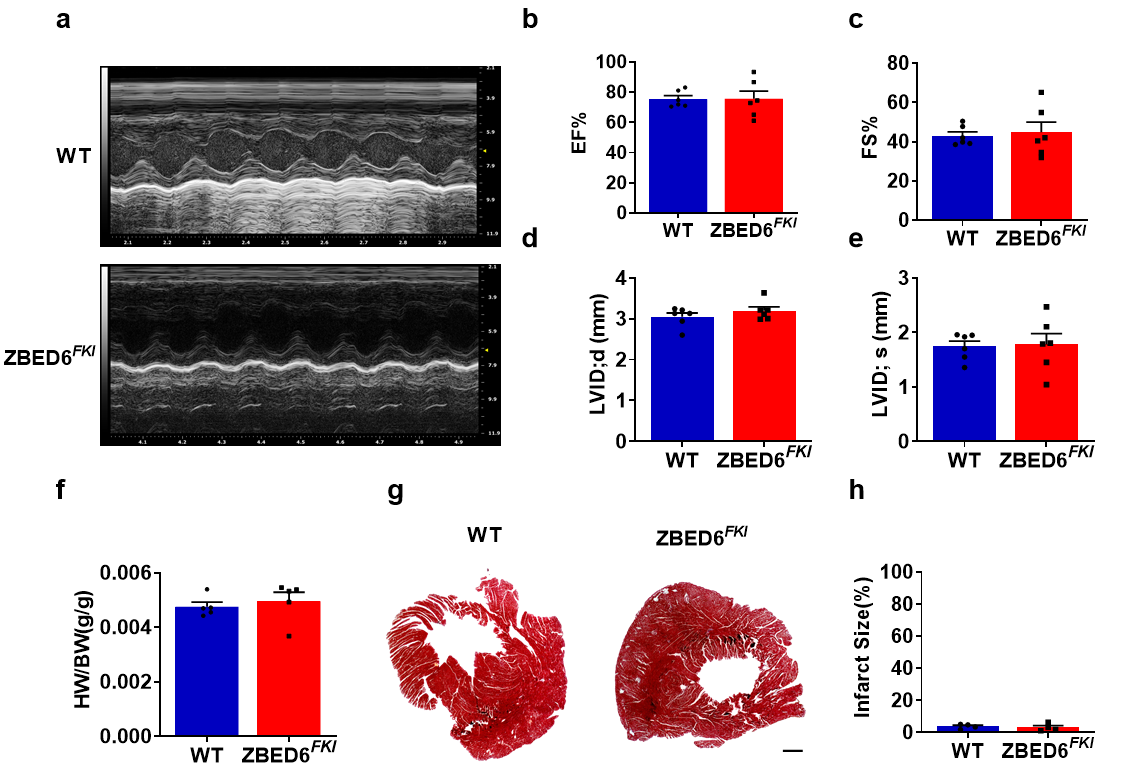
 Fig. S3.** The heart function of wild-type mice was not affected. **a** Echocardiography images and quantitative statistics. **b** EF (%), **c** FS (%), **d** LVID; d (mm) and **e** LVID; s (mm). *n* = 6. **f** Heart weight to body weight ratio. *n* = 5. **g** and **h** The areas of fibrosis in the infarcted hearts were assessed following Masson staining. Scale bar = 1 mm. *n* = 4.

*^
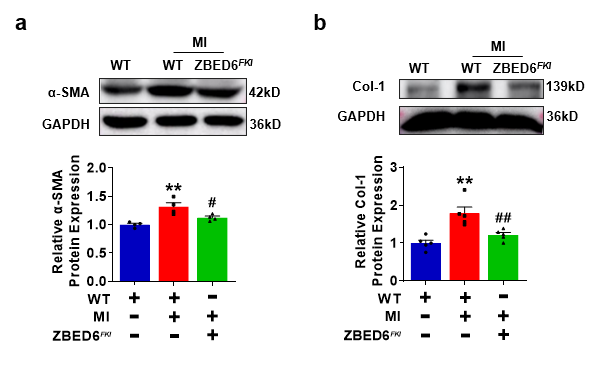
^*

**Fig. S4.** The protein expression of fibrotic genes. **a** The protein expression level of α-SMA was detected. *n* = 4. ^**^*P* < 0.01 *vs*. WT; ^#^*P* < 0.05 *vs*. WT+MI. **b** The protein expression level of Col-1was detected by Western Blot . *n* = 5. ^**^*P* < 0.01 *vs*. WT; ^##^*P* < 0.01 *vs*. WT+MI.

*^
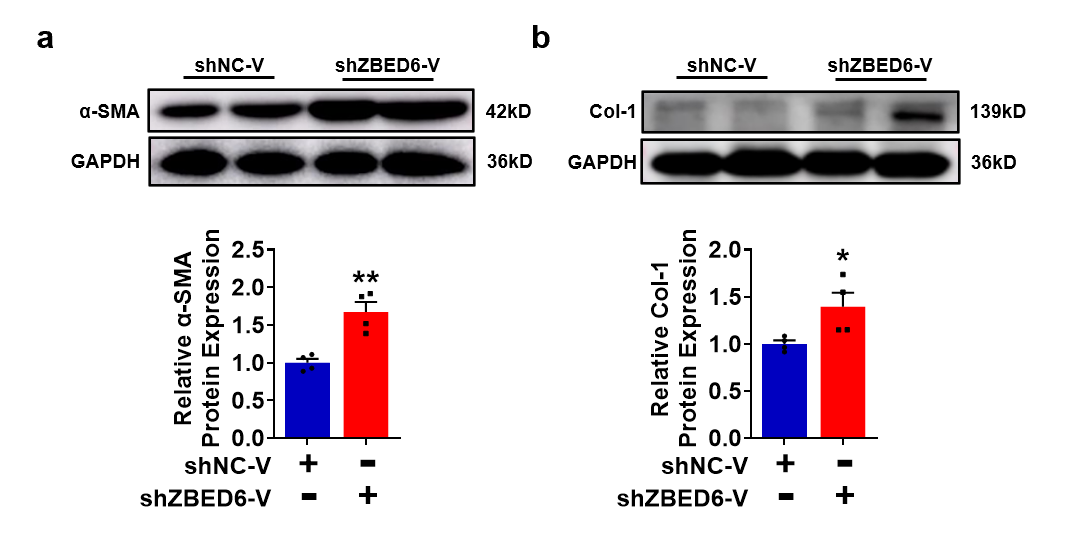
^*

**Fig. S5.** The protein expression of fibrotic genes. **a** The protein expression level of α-SMA was detected. *n* = 4. ^**^*P* < 0.01 *vs*. shNC-V. **b** The protein expression level of Col-1was detected by Western Blot . *n* = 4. ^*^*P* < 0.05 *vs*. shNC-V.

*^
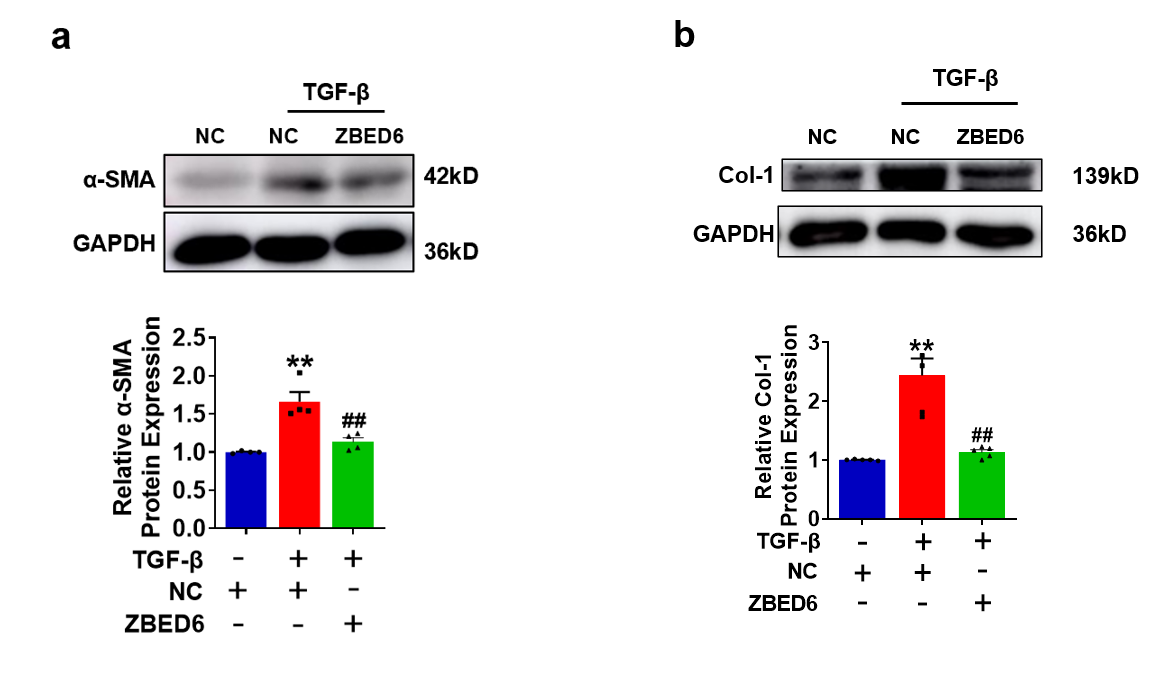
^*

**Fig. S6.** The protein expression of fibrotic genes. **a** The protein expression level of α-SMA was detected. *n* = 4. ^**^*P* < 0.01 *vs*. NC; ^#^*P* < 0.05 *vs*. NC+TGF-β. **b** The protein expression level of Col-1was detected by Western Blot . *n* = 5. ^**^*P* < 0.01 *vs*. NC; ^##^*P* < 0.01 *vs*. NC+TGF-β.


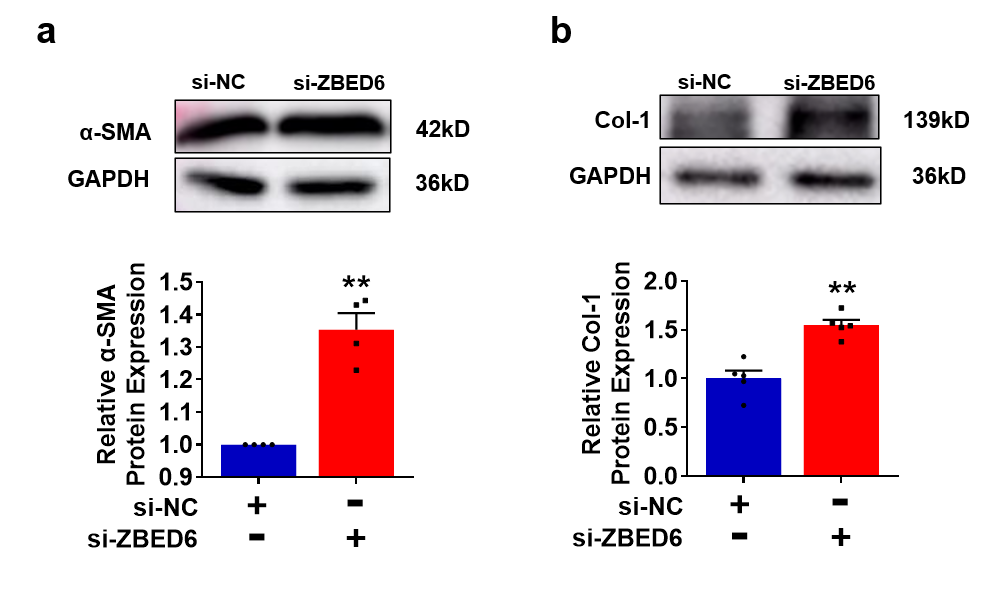


**Fig. S7.** The protein expression of fibrotic genes. **a and b** The protein expression level of α-SMA and Col-1 was detected. *n* = 4-5. ^**^*P* < 0.01 *vs*. si-NC.

**
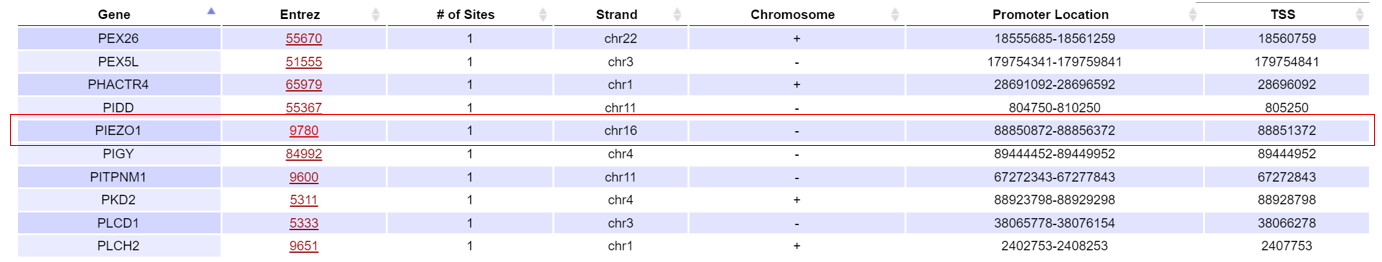
**

**Fig. S8.** Transcription factor prediction by Transcription Factor Target Gene Database (TFBS - Home (systemsbiology.net))

**
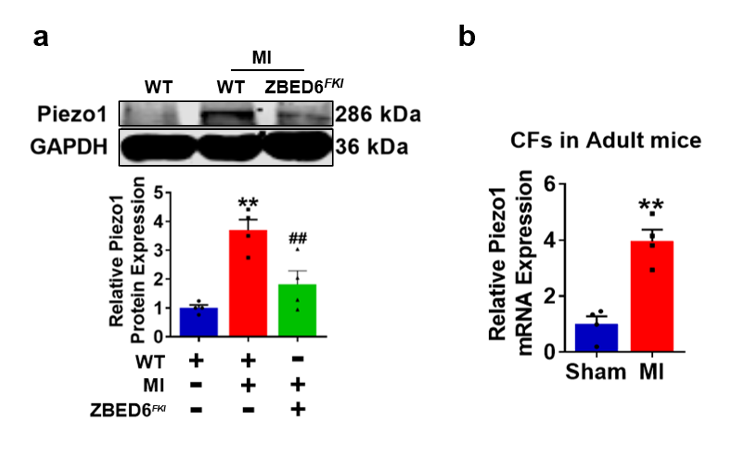
**

**Fig. S9.** Determination of Piezo1 protein expression. **a** The protein expression level of Piezo1 was detected. *n* = 4. ^**^*P* < 0.01 *vs*. WT; ^##^*P* < 0.05 *vs*. WT+MI. **b** The mRNA expression level of Piezo1 in CFs obtained from adult mice was detected. *n* = 4. ^**^*P* < 0.01 *vs*. Sham.

**Table S1:** The primer sequences employed for qPCR analysis are listed in Table S1.

| Gene | | Sequence (5’to 3’) |
| --- | --- | --- |
| GAPDH | F | AAGAAGGTGGTGAAGCAGGC |
|  | R | TCCACCACCCTGTTGCTGTA |
| 18S | F | CCTGGATACCGCAGCTAGGA |
|  | R | GCGGCGCAATACGAATGCCCC |
| ZBED6 | F | CTATCTGCCTAGTACCAGAGCC |
|  | R | CCTCCTCCCCATTAGTGACTC |
| Acta2 | F | GACGCTGAAGTATCCGATAG |
|  | R | CCACACGAAGCTCGTTATAG |
| Col1a1 | F | GCTCCTCTTAGGGGCCACT |
|  | R | CCACGTCTCACCATTGGGG |
| Col3a1 | F | ACGTAGATGAATTGGGATGCAG |
|  | R | GGGTTGGGGCAGTCTAGTG |
| Piezo1 | F | TGAGGCCTCCCATACCATACT |
|  | R | TGAGGCCTCCCATACCATACT |

Supplementary file 1 All genes detected in the RNA-sequence.
